# The Internet Hospital Plus Drug Delivery Platform for Health Management During the COVID-19 Pandemic: Observational Study

**DOI:** 10.2196/19678

**Published:** 2020-08-06

**Authors:** Liang Ding, Qiuru She, Fengxian Chen, Zitong Chen, Meifang Jiang, Huasi Huang, Yujin Li, Chaofeng Liao

**Affiliations:** 1 Clinical Trial and Research Center, People’s Hospital of Baoan Shenzhen Shenzhen China; 2 Department of Pharmacy, People’s Hospital of Baoan Shenzhen Shenzhen China

**Keywords:** internet hospital, drug delivery, internet hospital plus drug delivery, IHDD, health management, COVID-19

## Abstract

**Background:**

Widespread access to the internet has boosted the emergence of online hospitals. A new outpatient service called “internet hospital plus drug delivery” (IHDD) has been developed in China, but little is known about this platform.

**Objective:**

The aim of this study is to investigate the characteristics, acceptance, and initial impact of IHDD during the outbreak of COVID-19 in a tertiary hospital in South China

**Methods:**

The total number of and detailed information on online prescriptions during the first 2 months after work resumption were obtained. Patients’ gender, age, residence, associated prescription department, time of prescription, payment, and drug delivery region were included in the analysis.

**Results:**

A total of 1380 prescriptions were picked up or delivered between March 2 and April 20, 2020. The largest group of patients were 36-59 years old (n=680, 49.3%), followed by the 18-35 years age category (n=573, 41.5%). In total, 39.4% (n=544) of the patients chose to get their medicine by self-pickup, while 60.6% (n=836) preferred to receive their medicine via drug delivery service. The top five online prescription departments were infectious diseases (n=572, 41.4%), nephrology (n=264, 19.1%), endocrinology (n=145, 10.5%), angiocardiopathy (n=107, 7.8%), and neurology (n=42, 3%). Of the 836 delivered prescriptions, 440 (52.6%) were sent to Guangdong Province (including 363 [43.4%] to Shenzhen), and 396 (47.4%) were sent to other provinces in China.

**Conclusions:**

The IHDD platform is efficient and convenient for various types of patients during the COVID-19 crisis. Although offline visits are essential for patients with severe conditions, IHDD can help to relieve pressure on hospitals by reducing an influx of patients with mild symptoms. Further efforts need to be made to improve the quality and acceptance of IHDD, as well as to regulate and standardize the management of this novel service.

## Introduction

### Background

As the third largest country in the world by area, China has 34 provincial regions, over 1.4 billion people, but only 10 million licensed physicians (2.2 for every 1000 people) in 2019, according to the National Bureau of Statistics of China [1]. Since the severe acute respiratory syndrome (SARS) epidemic in 2003, the Chinese government has been rebuilding the three-tier health care system. Today, the health care system in China consists of community health centers (CHCs), and secondary and tertiary hospitals in urban areas; and village clinics, township health centers (THCs), and county hospitals in rural areas. CHCs, village clinics, and THCs are considered core primary care providers and are expected to provide affordable first contact care while secondary and tertiary care facilities provide specialist referral services [2]. However, with no gatekeeping in the primary health care system, patients can freely choose their provider at any health facility. Although many disorders could be treated by primary care providers conveniently and at a relatively low price, many patients are unwilling to see these providers owing to their lack of confidence in the health professionals’ skills and the quality of health care provided. They tend to go to high-level hospitals even for mild symptoms, effectively overcrowding those hospitals [3]. On the other hand, skilled doctors are unwilling to work at the community level and in remote rural areas for financial and professional reasons. These two problems have led to countless transprovincial patients, resulting in numerous additional economic and time costs [4].

The rapid increase in internet users (from 22.7% to 59.6% of the population between 2008 and 2018) [5] offers the Chinese government a new alternative to address these health care problems. On October 25, 2014, the first officially approved “internet hospital” went online in Guangdong Province. In the beginning, the internet hospital consisted of four clinics operated by doctors from the Second People’s Hospital of Guangdong Province, an online platform operated by a medical technology company, and a network of medical consulting facilities based in clinics in rural villages, CHCs, and large pharmacy chain stores [4]. The inchoate platform usually required onsite equipment (computers, cameras, speakers, and cable network). Patients needed to go to a medical consultation facility near their home and meet through the internet with the doctor based in a top-level hospital in a big city. With the widespread adoption of smartphones and tablet computers, and the ever-increasing popularity of mobile internet communication, a mobile health (mHealth) care model was made accessible to the public. mHealth allows patients to access information, assessments, and treatments in a timely manner. In addition, it empowers doctors with another way to connect with their patients and to practice without geographical limitations [6]. Therefore, the extra costs of health care, such as those associated with travel, time, and doctor consultations, can be dramatically reduced.

During the outbreak of coronavirus disease (COVID-19) [7], the Chinese government adopted a series of administrative measures to stop the spread of the epidemic [8], including requiring domestic internet hospitals to vigorously carry out remote medical services [9]. Although the convenience and ubiquity of internet hospitals makes them a promising avenue through which to overcome geographical limitations between patients and doctors, there is still a “social distance” barrier between the patients and their prescription medicines. In order to solve this problem, many hospitals intend to cooperate with delivery companies to build a partnership for drug delivery [10]. This bundled approach could offer an omnichannel solution that can help people on their path to urgently needed health care and medicine during the epidemic.

### Objective

To explore the advantages of the IHDD model for health management during public health emergencies, we analyzed the prescriptions of online outpatients at the People’s Hospital of Baoan Shenzhen in Shenzhen City, Guangdong. Data from the first 2 months after work resumption were collected to reveal the characteristics, acceptance, and initial impact of the new bundled approach.

## Methods

### Data Sources

The total number of online prescriptions and detailed information on them were obtained automatically from the hospital information system (DTHealth, V7.0) of the People’s Hospital of Baoan Shenzhen. Data from March 2 to April 20, 2020 were collected. Patients’ gender, age, residence, associated prescription departments, time of prescription, payment, and drug delivery region were included in the analysis. GraphPad Prism 8.0.2 (GraphPad Software) was used to summarize and analyze the data.

### Ethics Statement

Ethics approval was obtained from the medical research ethics committee of the People’s Hospital of Baoan Shenzhen before the start of the study.

## Results

### The Internet Hospital Workflow

**Figure 1:**
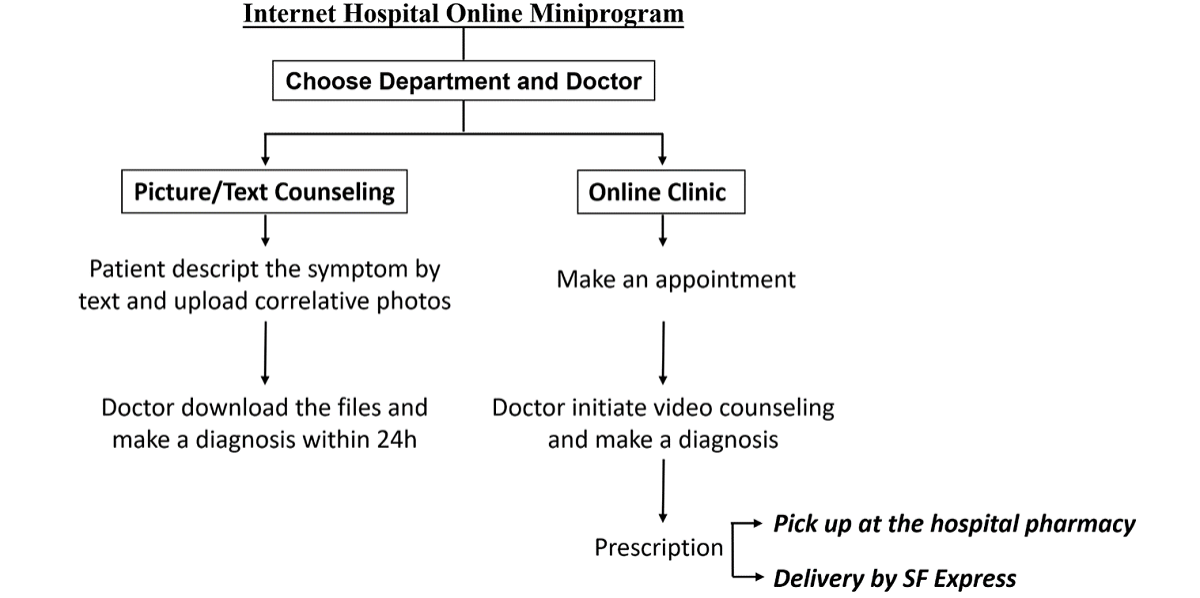
Online workflow of the internet hospital.

Figure 1 shows the online consultation workflow of the internet hospital. The patients chose a department and doctor by self-assessment using the hospital miniprogram. There was no prescription option for picture/text counseling patients. For the online clinic, a confirmation would be sent by text message once the online clinic appointment was made. Another message would be sent to the patient 3 minutes before the video counseling session began to remind them to open the hospital miniprogram in time. The doctor then initiated a video consultation with the patient and made an online prescription, if necessary, based on the diagnosis of the patient. At the payment step, the patient could choose self-pickup of medication at the hospital’s pharmacy or delivery to an assigned place.

### Number and Payment Amount of Online Prescriptions

**Figure 2:**
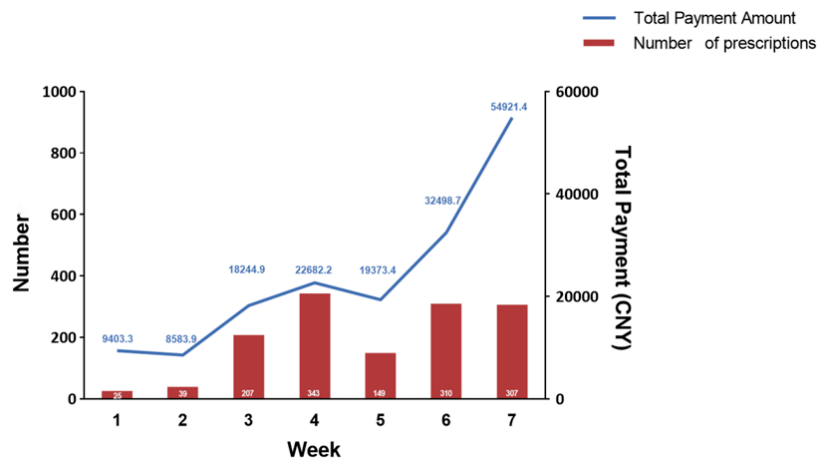
The number of and total payments pertaining to online prescriptions.

A total of 1380 prescriptions were picked up or delivered between March 2 and April 20, 2020. The weekly number and payment amount pertaining to these prescriptions are summarized in Figure 2. There was an increase in the use of the online prescription service. The number and total payments of the 7th week significantly increased, by 11.3 and 4.8 times, respectively, compared with the first week.

### Patient Characteristics

There was no sex-based differences among the patients who received prescriptions (Table 1). The patients were divided into four groups according to their age: 1-17 years old, 18-35 years old, 36-59 years old, and ≥60 years old. The largest group of patients were 36-59 years old (n=680, 49.3%), followed by those who were 18-35 years old (n=573, 41.5%). In total, 65.7% (n=907) of the patients were local residents, 5.6% (n=77) were from Guangdong cities other than Shenzhen, 28.7% (n=396) were from other provinces in China. Less than half the patients (n=544, 39.4%) chose to receive their medicine by self-pickup, while 60.6% (n=836) preferred to get their medicine by drug delivery service.

**Table 1:** Baseline characteristics of patients (N=1380).

Characteristic			Value
**Sex, n (%)**			
	Male		693 (50.2)
	Female		687 (49.8)
**Age (years)**			
	Median (range)		38 (1-93)
	**Group, n (%)**		
		1-17	12 (0.9)
		18-35	573 (41.5)
		36-59	680 (49.3)
		≥60	115 (8.3)
**Residence, n (%)**			
	Local (Shenzhen City)		907 (65.7)
	Other cities in Guangdong Province		77 (5.6)
	Other provinces		396 (28.7)
**Access to medicine, n (%)**			
	Delivery		836 (60.6)
	Self-pickup		544 (39.4)

### Distribution of Online Prescriptions

The top five online prescription departments were infectious diseases (n=572, 41.4%), nephrology (n=264, 19.1%), endocrinology (n=145, 10.5%), angiocardiopathy (n=107, 7.8%), and neurology (n=42, 3%). The majority of infectious disease and neurology patients chose drug delivery services, while most patients with other diagnoses preferred to pick up their medication (Table 2).

**Table 2:** Delivery/self-pickup preference of online prescription patients (N=1380).

Department	Delivery (n=836), n (%)	Self-pickup (n=544), n (%)
Infectious disease (n=572)	551 (96.3)	21 (3.7)
Nephrology (n=264)	86 (32.6)	178 (67.4)
Endocrinology (n=145)	48 (33.1)	97 (66.9)
Angiocardiopathy (n=107)	37 (34.6)	70 (65.4)
Neurology (n=42)	25 (59.5)	17 (40.5)

### Drug Delivery Details of Online Prescriptions

**Figure 3:**
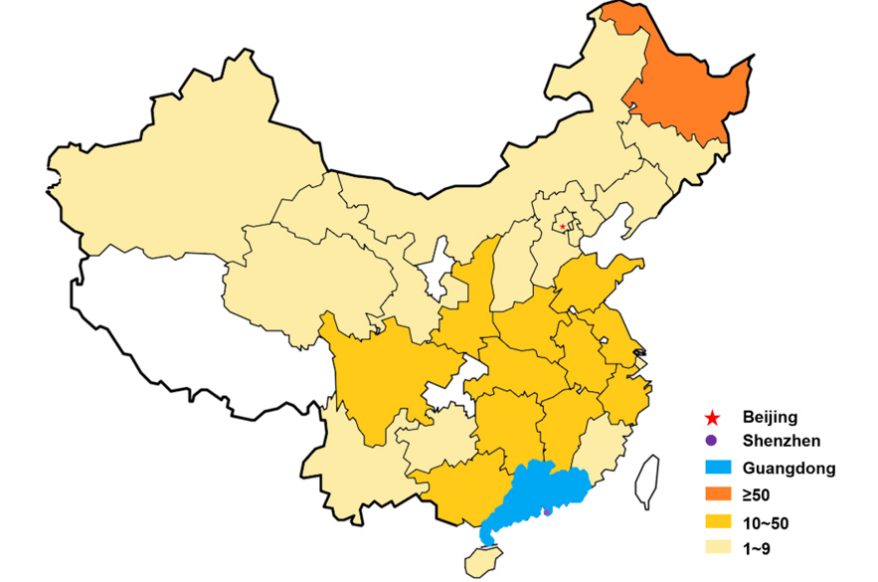
Regional distribution of prescription deliveries.

For the 836 delivered prescriptions, 440 (52.6%) were sent to Guangdong Province (including 363 [43.4%] to Shenzhen) and 396 (47.4%) were sent to other provinces in China. The top 10 provinces for out-of-province deliveries were Heilongjiang, Hubei, Guangxi, Shandong, Jiangsu, Hunan, Shanxi, Henan, Anhui, and Jiangxi (Table 3). Most of them are located in the northeast, eastern, and central parts of China (Figure 3 and Table 3). The top 10 delivered medicines are listed in Table 4. Most of the medicines were used to treat infectious or chronic disease, which was consistent with the distribution of online prescription.

**Table 3:** Out-of-province delivery details based on the geographical regions of China.

Region		Patients (n=396), n (%)
Northeast		107 (27.0)
Eastern		102 (25.8)
Central		82 (20.7)
Southern		35 (8.8)
Northwest		35 (8.8)
Northern		21 (5.3)
Southwest		14 (3.5)
**Top 10 provinces**		
	Heilongjiang	94 (23.7)
	Hubei	36 (9.1)
	Guangxi	33 (8.3)
	Shandong	27 (6.8)
	Jiangsu	24 (6.1)
	Hunan	24 (6.1)
	Shanxi	24 (6.1)
	Henan	22 (5.6)
	Anhui	14 (3.5)
	Jiangxi	14 (3.5)

**Table 4:** Top 10 delivered medicines.

Name	Formulation	Manufacturer
Entecavir	Dispersible tablet	ChiaTai TianQing
Metoprolol succinate	Sustained release tablet	AstraZeneca AB
Entecavir	Dispersible tablet	Dawnrays
Metformin hydrochloride	Tablet	Bristol-Myers Squibb
Tenofovir disoproxil fumarate	Tablet	Brilliant
Atorvastatin calcium	Tablet	Pfizer
Nifedipine	Controlled release tablet	Bayer
Atorvastatin calcium	Tablet	JiaLin
Mecobalamin	Tablet	Desano
Amlodipine besylate	Tablet	Pfizer

## Discussion

### Principal Findings

We conducted a pilot evaluation of the IHDD health care model in a tertiary hospital in Shenzhen during the first 2 months after work resumption. The unbalanced distribution of medical resources and the outbreak of COVID-19 [11,12] promoted the growth and exploration of more convenient internet-based medical practices [13], especially in the well-developed southern and southeastern parts of China, where people use the internet more often for medical purposes [14]. Despite the advantages of internet hospitals, access to medication remained an obstacle that may have discouraged people from using this platform. The traditional internet hospital required patients to go to the hospital or drugstore for medicines, which could cause more infections during an epidemic. The drug pickup process may increase risk of acute infectious disease, particularly for patients with suppressed immune systems or disabilities, which can then lead to severe health deterioration. On the other hand, out-of-city and out-of-province patients could have problems finding the exact prescription medications they need, as those medicines might be not available at their local hospitals and drugstores. In January 2019, the General Office of the State Council of the People's Republic of China implemented the National Centralized Drug Purchasing (NCDP) and Using pilot program and selected 11 cities (including Shenzhen) in mainland China to carry out the “4+7” City-Drug-Volume-Based-Purchasing pilot project [15]. As the “frontier” of Chinese prescription medicine reform, prescription medicines that were made by the doctors of Shenzhen’s hospitals may be more affordable. Therefore, the development of IHDD could enable patients across the country to access online prescription medication in a secure and convenient way.

The Chinese government has encouraged internet hospitals to join the epidemic prevention and control efforts of the COVID-19 outbreak [16]. On March 15, 2020, the first professional standard, “Specification for Online Consultation Service for Infectious Disease Epidemic Situation” was published on the national group standard information platform of China, requiring that internet hospitals provide 24/7 online services in response to the epidemic [17]. The internet hospital of the People’s Hospital of Baoan Shenzhen has been officially online since March 2, 2020. From opening to April 20, 2020, it saw a total of 8638 patients, an average of 176 per day, with 5877 in picture/text counseling and 2761 in online clinic video counseling (including 1381 that did not result in a prescription). Most of the picture/text consultations were prehospital services such as psychological counseling and medical education. The number and payment amounts of online prescriptions increased progressively from the first investigated week to the 7th one (Figure 2), which indicates increased acceptance of IHDD. The drop in prescription numbers during the 5th week might be the result of two factors: Tomb-Sweeping holiday and a lack of antiviral medicine.

Most prescription patients were between the ages of 18 and 60 years, had no time for onsite visitations, and had greater access to new medical platforms. At present, health authorities and the government have warned older people that they are at a higher risk of more serious and possibly fatal illness associated with COVID-19. Moreover, the global recommendation for older populations includes social isolation, which involves staying at home and avoiding contact with other people for an extended period of time [18]. Our data show that only 8.3% of IHDD users were ≥60 years (Table 1). This may be due to differences in public acceptance. Older populations usually take more time to become familiarized with the operations of IHDD.

The stay-at-home order constrained people from going outside, which increased difficulties associated with health management, especially chronic disease management. Medical professionals at hospitals with fever clinics are required to participate in COVID-19 prevention, control, and treatment, which has reduced their concentration on other diseases. In fact, the management of chronic disease has become a crucial issue in cities with large outbreaks of COVID-19 [19]. The largest number of internet hospital prescriptions came from the department of infectious diseases, which includes acute and chronic viral hepatitis, fatty liver, alcoholic hepatitis, drug-induced liver damage, autoimmune liver disease, and genetic and metabolic liver disease. The second largest group was from nephrology, followed by endocrinology. Patients with chronic liver disease, kidney disease, or diabetes could easily renew their prescriptions and receive their medicine by IHDD. A notable finding was that most patients with an infectious disease chose to receive their medicine by delivery (96.3%), whereas most patients with other diseases selected self-pickup (Table 2). This can be explained by the need for special storage of some medicines (eg, recombinant human erythropoietin for kidney disease or insulin for diabetes).

Established in 1984, the People’s Hospital of Baoan Shenzhen is also the Eighth People's Hospital of Shenzhen, the Shenzhen Baoan Affiliated Hospital of Southern Medical University, and the Second Affiliated Hospital of Shenzhen University. It was recognized as a Grade A Tertiary Hospital by the Guangdong Health Department in 2012. As an important source of health care providers in Shenzhen, the hospital holds a great reputation in both basic clinical practice and diverse clinical research and training. The IHDD platform enabled patients all over the country to obtain access to its health professionals and quality-assured medicine (Figure 3). The hospital even offered medical service to the patients in Wuhan, the epicenter of COVID-19. In fact, online prescription medicines that were delivered to Hubei Province accounted for the second largest number of all out-of-province deliveries (Table 3). The top 10 provinces for IHDD delivery were located in the northeast, eastern, and central parts of China. One of the reasons is that these areas are relatively economically developed regions, and their residents are highly educated, which ensures they have a better understanding of the benefits of IHDD.

The top 10 delivered medicines were used for the treatment of hepatitis, hypertension, hyperlipidemia, diabetes, climacteric symptoms, etc (Table 4). This finding was consistent with the distribution of prescription departments. In fact, 7 of these departments were enrolled in the National Essential Drugs of China program. Within these 7, 3 belonged to the “4+7” NCDP catalog. The affordability and quality of these medications were guaranteed by the government.

One of the concerns of IHDD is the safety and security of drug delivery. Therefore, delivery service companies with good reputations were chosen by the hospitals as partners. As the industry leader, SF Express is the first logistics company to cooperate with both pharmaceutical providers and hospitals. It has a long history of ambient temperature and cold chain medicine transport. Moreover, it offers real-time package tracking and zero-touch delivery. Once the patients place their order, a tracking number is sent by text message to their cell phone. The processing information of the medicine is updated and sent to patients automatically. Couriers place the medicines into the customer-assigned delivery lockers, which are usually near the patient’s residence, so the patient can access them using a random cipher. This process could effectively reduce viral transmission while simultaneously providing convenience for patients. At present, an advanced cooperation model is under exploration: the pharmacist-audited prescription will be sent to the manufacturer directly, and the medicines will be delivered from manufacturer’s stock house. This will reduce substantial pressure on hospital drugstores and cut transportation expenses.

### Future Prospects

Although in-person visits are essential when the patients are experiencing severe symptoms, IHDD can help to relieve pressure on hospitals by reducing the influx of mild cases. To make better use of IHDD during and after the current epidemic, more effort is needed. Simple and clear instructions are necessary to improve its acceptance by older people. Financial support, like adding medical insurance to payment methods, can also promote adoption by the public. The new hospital-manufacturer-patient transport model should be further evaluated. Moreover, official regulations are required in terms of standardization of the operational process and management of IHDD.

### Conclusion

The pandemic of COVID-19 has clearly entered a new stage with rapid spread to countries outside China, becoming a global threat. This once-in-a-century pandemic might permanently change people’s lifestyle, especially when it comes to health management. In our study, IHDD has been proven to be efficient and convenient for many types of patients during the crisis. The widespread use of this platform can help to reduce person-to-person transmission as well as the infection risk of patients with chronic diseases or disability.

## Abbreviations

CHC: community health center
COVID-19: coronavirus disease
IHDD: internet hospital plus drug delivery
mHealth: mobile health
NCDP: National Centralized Drug Purchasing
SARS: severe acute respiratory syndrome
THC: township health center
